# Gastric Neuroendocrine Tumor With Cystic Hepatic Metastases Mimicking Hepatic Echinococcosis: A Case Report

**DOI:** 10.7759/cureus.54507

**Published:** 2024-02-20

**Authors:** Rahul Gupta, Deepak Gusain, Nalini Bansal, Rahul Varshney, Arvind Singh

**Affiliations:** 1 Gastrointestinal Surgery, Synergy Institute of Medical Sciences, Dehradun, IND; 2 Pathology, SGT (Shree Guru Gobind Singh Tricentenary) University, Gurugram, IND; 3 Anesthesia and Critical Care, Synergy Institute of Medical Sciences, Dehradun, IND; 4 Gastroenterology, Synergy Institute of Medical Sciences, Dehradun, IND

**Keywords:** cystic hepatic lesions, hepatic cyst differential, hepatic echinococcosis, malignant gastric ulcer, severe hematemesis, hepatic metastases, gastric neuroendocrine tumors, left hepatectomy

## Abstract

Metastatic gastrointestinal neuroendocrine tumors classically appear as contrast-enhancing lesions on computed tomography. However, in a small percentage of patients, these lesions can be cystic in nature, leading to false diagnoses of benign or infectious lesions such as echinococcosis. Hence, every cystic lesion of the liver must be carefully investigated before making the treatment plan. We report a patient with hematemesis caused by a large gastric ulcer with multiple cystic lesions in the left lobe of the liver abutting the stomach. The liver lesions were misdiagnosed as hepatic echinococcosis, and the patient was started on medical therapy. However, when medical therapy failed, the patient underwent surgical excision and the histopathology showed cystic metastases of a gastric neuroendocrine tumor.

## Introduction

Cystic lesions of the liver are commonly encountered in abdominal imaging. The differential diagnoses vary from benign simple cysts to potentially life-threatening cancers. They are classified into developmental, inflammatory, neoplastic, and trauma-related lesions based on their etiology [1]. Benign cystic hepatic lesions are very common in clinical practice. On the other hand, malignant hepatic cysts are rare and require a high index of clinical and radiological suspicion for their preoperative diagnosis. Abdominal ultrasound can accurately diagnose simple hepatic cysts and polycystic liver disease while contrast-enhanced computed tomography (CT) and magnetic resonance imaging (MRI) are required to determine the exact nature of the hepatic cysts. However, in some cases, even CT and MRI may not be able to differentiate between benign and malignant hepatic cysts due to overlapping features [2-4]. Here, we report a patient with a cystic hepatic neuroendocrine tumor (NET) mimicking hepatic echinococcosis (HEC) on preoperative imaging. The definitive diagnosis was made based on the histological examination of the resected specimen.

## Case presentation

A 53-year-old male presented to the emergency department with hematemesis and melena for three days. There was no history of similar complaints in the past. The patient denied having any addictions. There was no significant medical, surgical, or family history. On physical examination, the patient was pale, non-icteric, and dehydrated. His pulse rate was 104 beats/min, blood pressure of 100/70 mmHg, and respiratory rate of 20 breaths/min. The per abdominal examination revealed an ill-defined, non-tender lump in the epigastrium with an irregular surface, firm in consistency, and cephalo-caudal movement with respiration. The patient was admitted to the intensive care unit and resuscitated with intravenous fluids.

**Figure 1:**
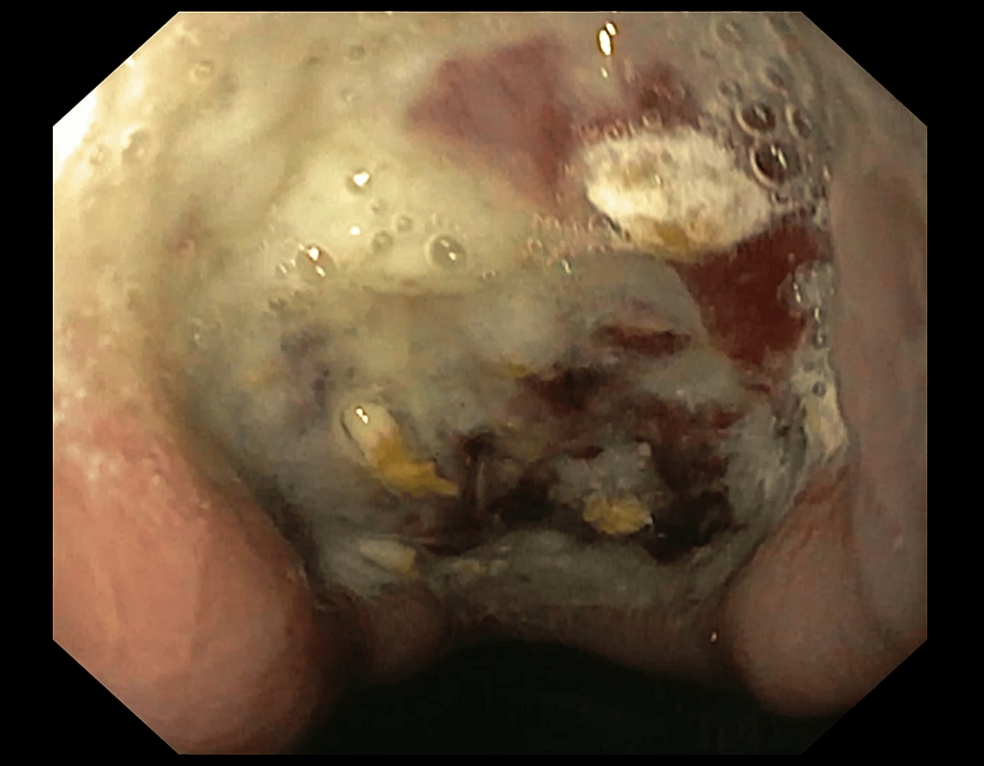
Esophagogastroscopy showing a large ulcer along the lesser curvature of the body of the stomach

Emergency esophagogastroscopy was performed, which revealed a large ulcer with an adherent clot along the lesser curvature in the gastric body (Figure 1). The rest of the stomach, esophagus, and duodenum were unremarkable. A biopsy was not taken, and a repeat endoscopy for a biopsy was planned after initial medical therapy. Blood investigations revealed anemia (hemoglobin 7.5 gm/dl).

**Figure 2:**
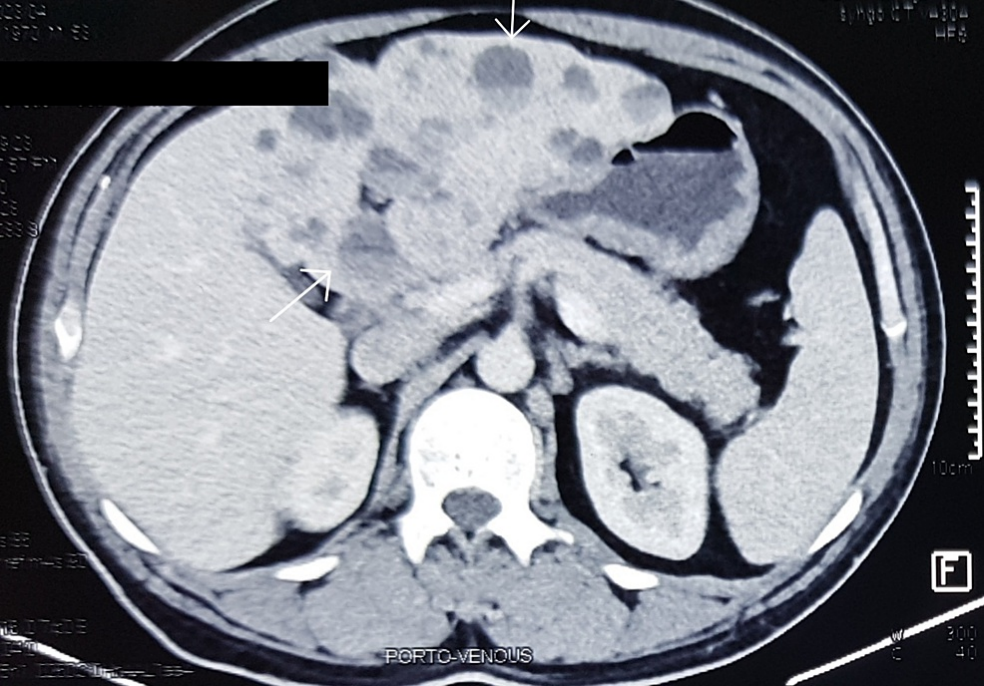
Contrast-enhanced computed tomography of the abdomen showing multiple cystic lesions on the left with fluid debris (arrow)

Contrast-enhanced CT abdomen showed hepatomegaly with multiple discrete and confluent cystic lesions of varying size involving segments II, III, IV, and VI (Figure 2). The lesions were round to ovoid containing fluid debris and daughter cysts with the largest cyst measuring 8.9 x 7.6 x 8.2 cm. Additionally, multiple peripherally located round to oval cysts were seen with the lesions suggestive of daughter cysts.

**Figure 3:**
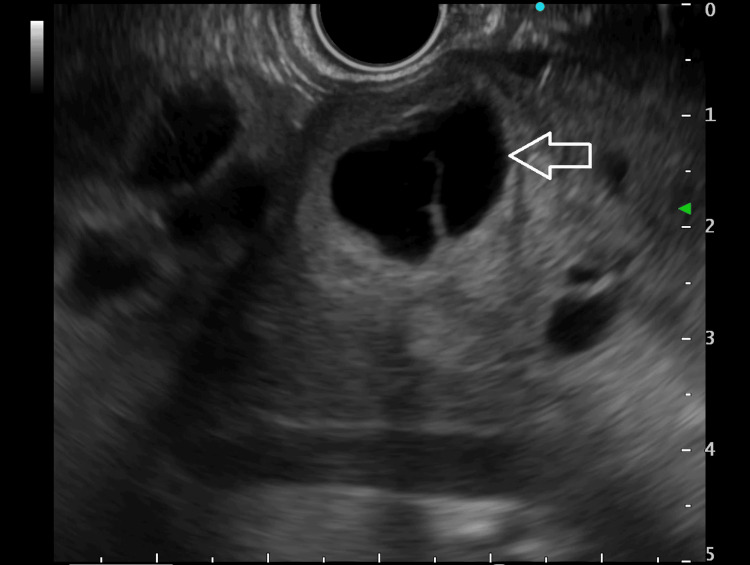
Endoscopic ultrasound showing multiple hypoechoic lesions (arrow) in the left liver lobe with thin septae and one of them abutting the gastric wall

Endoscopic ultrasound (EUS) found multiple cysts in the left lobe of the liver with membranes and daughter cysts suggestive of hepatic echinococcosis (HEC) (Figure 3). One of the cysts was seen breaching the gastric mucosa at the ulcer site. Based on the above findings, the provisional diagnosis of gastric ulcer with HEC was made. Hydatid serology was negative.

**Figure 4:**
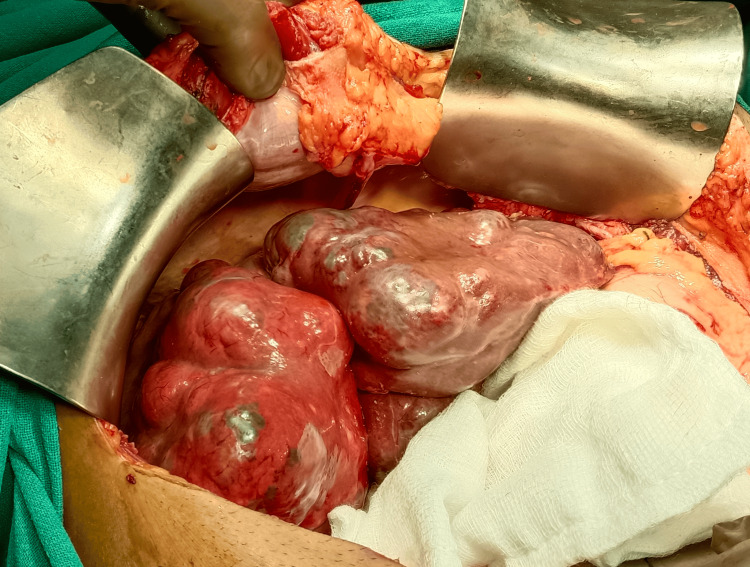
Intraoperative picture showing the left liver studded with multiple cysts of varying sizes

The patient was started on proton pump inhibitors, tranexamic acid for the gastric ulcer, and oral albendazole 400 mg twice a day for HEC. However, the patient developed rebleeding after three days requiring a blood transfusion. In view of the failure of medical therapy, the patient was planned for emergency surgery. The patient was operated on under general anesthesia using a bilateral subcostal incision. At surgery, the left lobe of the liver was found to be enlarged up to 30 cm with the presence of multiple cystic lesions varying in size from 1 cm to 6 cm in diameter (Figure 4).

**Figure 5:**
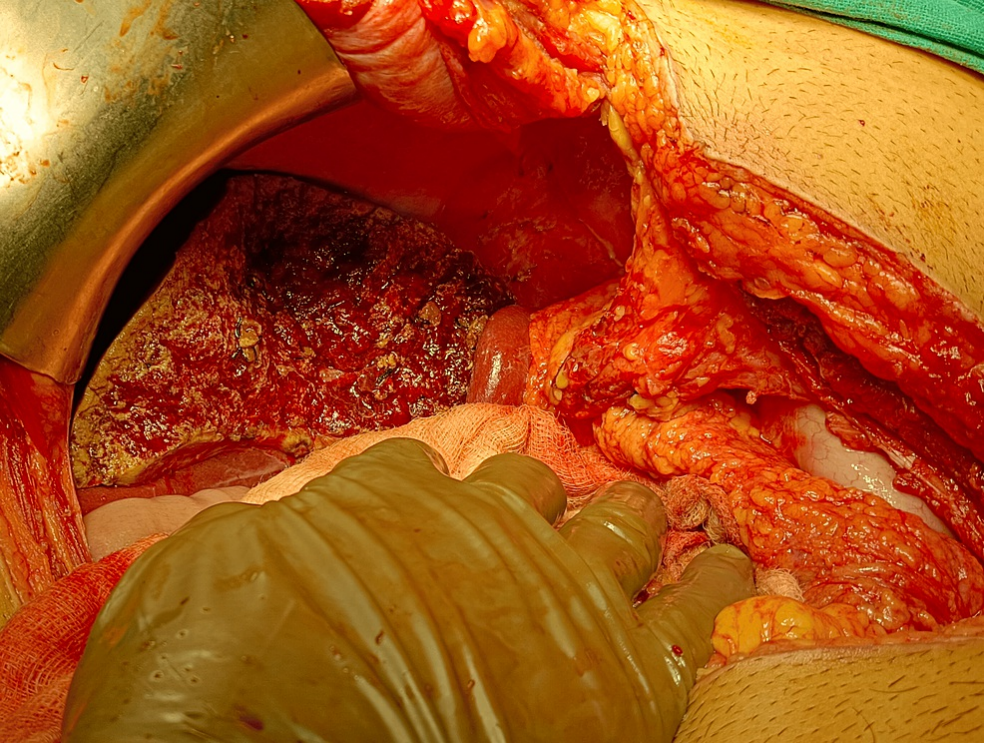
Intraoperative picture showing the cut surface of the liver after left hepatectomy.

The cystic lesions were reaching up to the gallbladder fossa. A single cyst of 2 x 2 cm was found in segment VI of the right lobe. Additionally, a hard mass of 4 cm x 4 cm was present in the lesser curvature of the gastric body with enlarged lymph nodes along the left gastric vessels. In view of the above findings, cholecystectomy, left hepatectomy, excision of the right lobe lesion, and stapled partial gastrectomy with adjoining lymphadenectomy were performed (Figure 5). The operative time was 360 minutes and the estimated blood loss was 700 ml. In the postoperative period, there was biliopurulent drain output, which was managed conservatively with oral antibiotics and regular flushing of the drain. The postoperative hospital stay was four days.

**Figure 6:**
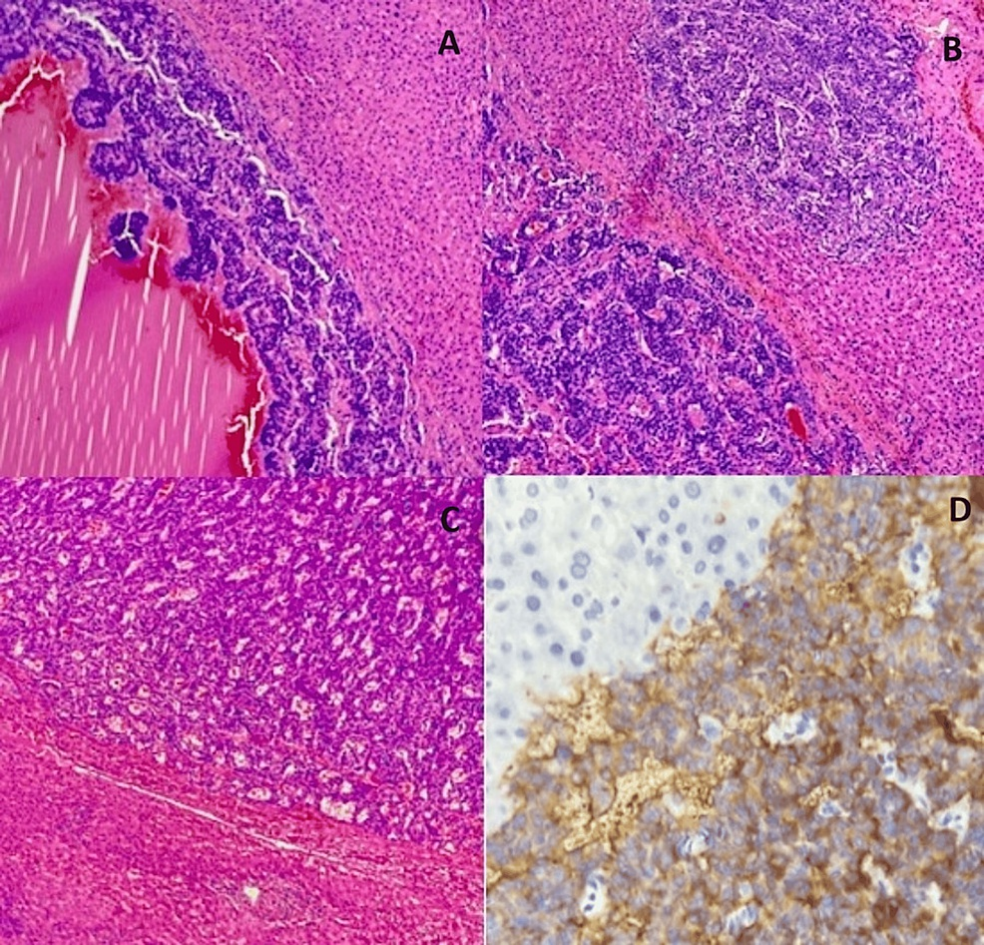
Microscopic examination (A) Blood-filled cyst lined by neoplastic cells (hematoxylin & eosin, 10x), (B) solid nests of tumor cells within the liver parenchyma (hematoxylin & eosin, 10x), (C) neuroendocrine tumor cells with adjacent compressed hepatic parenchyma (hematoxylin & eosin, 20x), (D) immunohistochemistry showed positive staining in tumor cells (synaptophysin, 40X).

The cut section revealed multiple small and large cystic spaces filled with blood and muddy-colored fluid. The larger cysts were multiloculated. Histopathological examination of the gastrectomy specimen revealed neoplastic cells arranged in nests with a trabecular, pseudo-glandular, and festooning pattern. The neoplastic cells had mildly pleomorphic nuclei with fine chromatin and moderate cytoplasm. The tumor cells were seen infiltrating up to the muscularis propria. Lymphovascular and perineural invasions were present. One out of seven lymph nodes showed metastatic deposits. The left hepatectomy specimen showed multiple blood-filled cysts with hemorrhagic spaces lined by neoplastic cells similar to that seen in the resected stomach (Figure 6). The tumor cells showed positive staining by synaptophysin. The adjacent liver parenchyma was unremarkable. All the resection margins were tumor-free. The final diagnosis was a type 3 metastatic gastric neuroendocrine tumor (pT2N1M1a). The patient was referred to a higher center for adjuvant therapy.

## Discussion

Neuroendocrine tumors (NETs) are rare types of gastrointestinal tumors accounting for 1-2% of all cases [5]. About 20-30% of patients have distant metastases at the time of diagnosis, with the most common site being the liver [6]. Hepatic NETs are mostly metastatic lesions from the primary gastrointestinal NETs. These hepatic lesions are typically solid lesions with intense contrast uptake on contrast-enhanced CT or MRI. Cystic hepatic NETs are very rare, with most of them being primary hepatic NETs [2,4,5]. The cystic appearance of hepatic lesions could be due to the high mucinous content within the lesions in diseases such as biliary cystadenomas, metastasis from ovarian carcinomas, or the rapid growth of the lesions causing hemorrhage, cystic degeneration, or necrosis as seen in the present case [1].

The clinical presentation of hepatic NET depends upon whether the NET is functional or non-functional. The functional lesions cause symptoms of Zollinger-Ellison syndrome, Cushing syndrome, or carcinoid syndrome depending upon the hormone production. These lesions are often small at the time of diagnosis due to prominent symptoms. However, non-functional hepatic NETs are often large by the time they give rise to non-specific symptoms of abdominal pain, fullness, or jaundice due to the mass effect. Patients with secondary hepatic NETs can also present with symptoms related to the primary gastrointestinal lesions as seen in the present case.

On contrast-enhanced CT or MRI, these lesions are characteristically solid in nature with intense contrast uptake. Rarely, they can be cystic in nature as seen in the index case. In the current case, there were multiple hepatic cystic lesions of varying size with fluid debris within them due to the hemorrhagic degeneration on CT mimicking hydatid sand, which led to the misdiagnosis of HEC. Similar findings were noted by Krohn et al. on MRI [2]. In such cases, it is important to differentiate them from benign hepatic lesions. The radiological features suggestive of malignant hepatic cystic lesions include the presence of complex cystic lesions with thick enhancing septations and the presence of solid areas [2]. MRI with hepatocyte-specific contrast material such as Gd-EOB-DTPA can also be used to differentiate benign from malignant cystic lesions of the liver [2,4]. Functional imaging modalities such as OctreoScan and Gallium-68 receptor positron emission tomography-computed tomography (PET-CT) are very useful for preoperative diagnosis and staging of NET [3,7]. In patients with poorly differentiated NET, 18-fluorodeoxyglucose PET-CT has been found to be more sensitive than these functional modalities [7]. Percutaneous biopsy must be considered in patients with unresectable disease and atypical radiological findings [4].

Surgical resection, if feasible, offers the best survival benefit to patients with primary or metastatic hepatic NET [8]. It also serves both diagnostic and therapeutic purposes in those with atypical findings as seen in the present case. In patients with unresectable disease with a preoperative diagnosis of metastatic hepatic NET, surgical debulking can be considered if more than 90% of the disease can be excised [8]. Other palliative therapies for patients with unresectable hepatic NET include trans-arterial chemoembolization (TACE), systemic chemotherapy, somatostatin analogs, and local therapies such as radiofrequency ablation. The prognosis depends upon the stage, grade, and location of primary NET [8,9].

## Conclusions

Cystic hepatic neuroendocrine tumors are rare. A high index of clinical and radiological suspicion is required to differentiate them from benign hepatic cysts such as HEC. Functional imaging, such as OctreoScan and Gallium-68 receptor PET-CT, can aid in making accurate diagnoses in patients with atypical radiological findings. Surgical resection is the treatment of choice for resectable hepatic NETs.
